# 供者特征对骨髓增生异常综合征患者单倍型移植预后的影响：一项回顾性队列研究

**DOI:** 10.3760/cma.j.cn121090-20241231-00605

**Published:** 2025-11

**Authors:** 虹 王, 学谦 李, 清源 汪, 嘉乾 戚, 惠英 仇, 琤琤 傅, 晓文 唐, 瞄 苗, 荧 王, 苏宁 陈, 长耿 阮, 德沛 吴, 悦 韩

**Affiliations:** 苏州大学附属第一医院，江苏省血液研究所，国家血液系统疾病临床医学研究中心，国家卫生健康委员会血栓及止血重点研究室，苏州 215006 The First Affiliated Hospital of Soochow University, Jiangsu Institute of Hematology, National Clinical Research Center for Hematologic Diseases, Key Laboratory of Thrombosis and Hemostasis of Ministry of Health, Suzhou 215006, China

**Keywords:** 单倍型造血干细胞移植, 供者选择, 骨髓增生异常综合征, 预后, Haplo-identical donor transplantation, Donor selection, Myelodysplastic syndrome, Prognosis

## Abstract

**目的:**

评估供者特征对骨髓增生异常综合征（Myelodysplastic syndrome, MDS）单倍型造血干细胞移植预后的影响。

**方法:**

回顾性分析2007年12月至2019年9月203例在苏州大学附属第一医院接受单倍型造血干细胞移植的MDS患者临床资料，评估供者特征对移植预后的影响。

**结果:**

供者年龄>50岁与移植后较高的EB病毒（EBV）激活风险相关，2年EBV激活累积发生率为42.9％，而供者年龄≤50岁的亚组其累积发生率为22.0％（*P*＝0.010）。女性供者组移植后2年重度慢性移植物抗宿主病（GVHD）的累积发生率为11.9％，显著高于男性供者组的4％（*P*＝0.017）。此外，女性供者移植后总生存（OS）率稍劣于男性供者，移植后2年OS率分别为56.6％和69.7％（*P*＝0.073）。供受者血型对移植后OS率和累积复发率无显著影响。供受者亲缘关系分析表明，子女供者与单倍型同胞供者或父母供者相比，移植后Ⅱ～Ⅳ度急性GVHD累积发生率更低（27.2％对45.7％对53.5％，*P*＝0.007），其2年EBV激活的累积发生率也更低（13.9％对29.3％对38.9％，*P*＝0.001）。当供者年龄<20岁时，性别对移植后2年OS（*P*＝0.913）、2年无复发生存（*P*＝0.716）及100 d内Ⅱ～Ⅳ度急性GVHD（*P*＝0.359）影响无统计学意义。

**结论:**

MDS单倍型造血干细胞移植中避免采用50岁以上的大龄供者。男性供者优于女性供者，子女单倍型供者优于同胞单倍型或父母单倍型供者。如果供者年龄小于20岁，性别对移植预后无显著的不良影响。

## Abstract (en)

**Objective:**

To evaluate the impact of donor characteristics on the prognosis of myelodysplastic syndrome（MDS）patients undergoing haplo-identical transplantation（HIDT）.

**Methods:**

A retrospective analysis of 203 MDS patients who received HIDT was conducted to evaluate how donor factors influenced transplant outcomes.

**Results:**

In MDS patients undergoing haploidentical transplantation, donors over 50 years were associated with higher EBV reactivation（2-year cumulative incidence 42.9％ *vs* 22.0％ for <50 years old; *P*＝0.010）. Female donors were linked to increased severe chronic GVHD compared with male donors（2-year incidence 11.9％ *vs* 4.0％; *P*＝0.017）. Additionally, 2-year overall survival（OS）was slightly lower with female donors than male donors（56.6％ *vs* 69.7％）, but the difference was not statistically significant（*P*＝0.073）. Donor-recipient blood type did not affect post-transplant OS or cumulative relapse rates. Donor-recipient kinship analysis revealed that child donors, compared to haploidentical sibling or parent donors, had lower rates of grade Ⅱ–Ⅳ acute GVHD（27.2％ *vs* 45.7％ *vs* 53.5％, *P*＝0.007）and 2-year EBV reactivation（13.9％ *vs* 29.3％ *vs* 38.9％, *P*＝0.001）. For donors under 20 years, donor gender did not significantly affect 2-year OS（*P*＝0.913）, relapse-free survival（*P*＝0.716）, or 100-day incidence of grade Ⅱ–Ⅳ acute GVHD（*P*＝0.359）.

**Conclusion:**

For MDS patients undergoing HIDT, donors over 50 should be avoided. Male and child donors are preferred, while donor gender does not significantly affect outcomes if the donor is under 20 years old.

异基因造血干细胞移植（allo-HSCT）是治疗中高危骨髓增生异常综合征（Myelodysplastic syndrome, MDS）的重要手段。HLA配型全相合同胞或无关供者仍是目前指南推荐的首要供者选择[1]。然而，仅有少数患者能及时获得全相合供者，因此单倍型移植应运而生，并凭借免疫抑制策略的发展在国内与国际上取得了重大进展[2]–[5]。现有证据表明，在急性白血病[6]–[7]和MDS[8]中，单倍型移植可取得与同胞全相合移植相似的疗效，已成为重要的替代治疗手段。

随着单倍型移植的广泛开展，如何优化供者选择以进一步改善预后，已成为新的临床焦点。尽管欧洲血液与骨髓移植学会（EBMT）发布了针对血液恶性肿瘤单倍型移植供者选择的指导建议[9]，但其建议更多是基于急性白血病的数据，并未针对MDS提供特异性的、证据充分的指导。MDS独特的疾病生物学特征及患者群体的老年化趋势，可能使得供者因素（如年龄、性别及亲缘关系）对其移植预后的影响模式不同于其他血液肿瘤。因此，在MDS单倍型移植领域，关于供者年龄的最佳界值、不同年龄层中性别匹配的效应差异、不同亲缘关系的优劣对比等问题，仍缺乏系统性的大样本数据支持。本研究中，我们回顾性分析单中心203例MDS患者移植资料，评估供者特征对移植预后的影响，以期为MDS单倍型移植的供者选择提供参考。

## 病例与方法

一、病例资料

本研究为回顾性队列研究。2007年12月至2019年9月，总计203例MDS患者在我院进行单倍型移植。本研究回顾性分析了患者临床疾病特征及供者特征，以评估供者特征对MDS移植预后的影响。收集的供者特征资料包括供者性别、供者年龄、供受者血型及HLA匹配程度、供受者亲缘关系。MDS患者临床资料包含年龄、性别、WHO分型、国际预后评分系统（IPSS）评分、修订的国际预后评分系统（IPSS-R）评分。MDS疾病诊断参考2016版WHO分型标准[10]。

二、预处理方案

所有患者均采用改良BuCy预处理方案，具体用药为：司莫司汀（250 mg/m^2^，−10 d），阿糖胞苷（4 g·m^−2^·d^−1^，−9～−8 d），白消安（3.2 mg·kg^−1^·d^−1^，−7～−5 d），环磷酰胺（1.8 g·m^−2^·d^−1^，−4～−3 d）。

三、移植物抗宿主病（GVHD）预防及评估

兔抗人胸腺细胞球蛋白（anti-thymocyte globulin，ATG）或ATG-F（Fresenius Biotech GmbH）用于体内去除T细胞以预防GVHD，具体用法：ATG为2.5 mg·kg^−1^·d^−1^，−5～−2 d；ATG-F为5 mg·kg^−1^·d^−1^，−5～−2 d。此外，所有患者均接受环孢素A、吗替麦考酚酯和短程甲氨蝶呤预防GVHD。移植后急性GVHD的分级标准参考《中国异基因造血干细胞移植治疗血液系统疾病专家共识（Ⅲ）——急性移植物抗宿主病（2020年版）》[11]。慢性GVHD分级标准参考《慢性移植物抗宿主病诊断与治疗中国专家共识（2021年版）》[12]。

四、巨细胞病毒（CMV）及Epstein-Barr病毒（EBV）预防、监测与治疗

移植前供者及患者均接受EBV-DNA、CMV-DNA及CMV IgG、IgM抗体检测。预处理过程所有患者接受更昔洛韦（5 mg/kg每12 h 1次，连用7 d）预防CMV活化。+180 d内EBV-DNA及CMV-DNA每周检测1～2次，+180 d后可根据具体情况延长检测间隔或停止常规检测。外周血CMV-DNA出现1次>1 000拷贝数/ml或两次>500拷贝数/ml开始经验性采用更昔洛韦或缬更昔洛韦或膦甲酸钠治疗。外周血EBV-DNA出现1次>1 000拷贝数/ml或两次>500拷贝数/ml，经验性采用利妥昔单抗清除EBV，同时密切关注有无移植后淋巴增殖性疾病（PTLD）相关症状及体征。

五、研究终点及定义

主要研究终点为总生存（OS）时间，定义为从单倍型移植到患者死亡或末次随访的时间间隔。本研究也对移植后复发、非复发死亡（non-relapse-mortality，NRM）、急慢性GVHD、CMV感染及EBV感染累积发生率进行了调查。复发定义为骨髓原始细胞比例>5％和（或）潜在疾病的重新出现。NRM定义为+28 d内任何原因死亡或在+28 d后无疾病复发迹象的死亡。

六、统计学处理

采用SPSS 23.0软件及R 4.5.1进行统计分析。采用Gray检验进行竞争风险事件累积发生率的计算与比较[13]，NRM与复发互为竞争因素，急慢性GVHD以非GVHD导致的死亡为竞争因素，EBV或CMV激活发生率以非EBV或CMV激活导致的死亡为竞争因素。采用Kaplan-Meier法进行OS的生存分析，组间比较采用Log-rank检验。双侧*P*<0.05为差异有统计学意义。

## 结果

一、基线特征及整体预后

203例患者中位年龄41（7～63）岁。根据2016 WHO分型，原始细胞增多（EB）-1 52例（26％），EB-2 85例（42％），难治性血细胞减少伴多系病态造血（RCMD）66例（32％）。MDS患者疾病特征及供者特征见表1。203例患者中，97.5％在移植后30 d内达到了供者完全嵌合（>95％）。中位随访28.2（0.2～117）个月，80例患者死亡，23例患者复发。2年OS率及NRM分别为（64.9±3.4）％、（28.1±3.2）％。移植后第100天Ⅱ～Ⅳ度急性GVHD的累积发生率为（40.4±3.8）％，移植后2年重度慢性GVHD的累积发生率为（10.8±2.6）％。

**表1:** 203例接受单倍型移植骨髓增生异常综合征（MDS）患者及其供者的临床特征

临床特征	例数	构成比（％）
MDS患者特征		
性别		
男	131	64.5
女	72	35.5
年龄（岁）		
<40岁	94	46.3
≥40岁	109	53.7
WHO分型		
MDS-EB-1	52	25.6
MDS-EB-2	85	41.9
MDS-RCMD	66	32.5
IPSS评分^a^		
低危/中危-1	100	49.5
中危-2/高危	102	50.5
IPSS-R评分^a^		
低危/中危	65	32.2
高危/极高危	137	68.8
MDS供者特征		
供者年龄		
<20岁	32	15.8
20～50岁	150	73.9
>50岁	21	10.3
供者性别		
男性	129	63.5
女性	74	36.5
供受者性别		
相同	111	54.7
男供男	84	41.4
女供女	27	13.3
不同	92	45.3
男供女	45	22.2
女供男	47	23.2
供受者ABO血型		
相合	113	55.7
次要不合	37	18.2
主要不合	39	19.2
主次均不合	14	6.9
HLA相合位点		
5/10	153	75.4
6～7/10	34	16.7
8～9/10	16	7.9
供者亲缘关系	
子女	108	53.2
父母	54	26.6
同胞或旁系	41	20.2

**注** MDS-EB：MDS伴原始细胞增多；MDS-RCMD：难治性血细胞减少伴多系病态造血；IPSS：国际预后评分系统；IPSS-R：修订的IPSS；HLA：人类白细胞抗原。^a^1例患者无染色体核型结果，因此202例患者有IPSS及IPSS-R评分数据

二、供者特征对MDS单倍型移植预后的影响

供者年龄、性别、血型、亲缘关系及HLA位点对移植预后的影响见表2。供者年龄≥50岁时，MDS患者移植后EBV激活累积发生率增加（*P*＝0.010）。与男性供者相比，女性供者组移植后重度慢性GVHD的累积发生率更高（*P*＝0.017），其整体OS也略低但差异无统计学意义（*P*＝0.073）。在不同供受者性别组合中，女供女组移植后重度慢性GVHD发生率最高。供受者血型对移植后OS和累积复发率无显著影响，但主要不合组患者移植后Ⅱ～Ⅳ度急性GVHD的累积发生率为（51.3±8.5）％，高于其他亚组但差异无统计学意义（*P*＝0.085）。供受者亲缘关系分析表明，与单倍型同胞供者或父母供者相比，子女供者移植后Ⅱ～Ⅳ度急性GVHD和EBV激活的累积发生率更低（*P*值分别为0.007、0.001），同时子女供者移植后OS率低于父母供者，但差异无统计学意义（*P*＝0.332）。

**表2:** 供者特征对骨髓增生异常综合征（MDS）患者单倍型移植各事件累积发生率的影响（％，*x*±*SE*）^a^

因素	例数	Ⅱ～Ⅳ度aGVHD	重度cGVHD	CMV激活	EBV激活	NRM	复发	OS
供者年龄								
<50岁	182	37.3±3.7	5.9±1.9	39.0±3.6	22.0±3.1	27.0±3.3	9.3±2.2	65.8±3.5
≥50岁	21	41.9±11.9	10.2±7.1	33.3±10.6	42.9±11.2	28.6±10.2	9.5±6.6	57.1±10.8

*P*值		0.878	0.150	0.809	0.010	0.879	0.276	0.298

供者性别								
男性	129	39.5±4.5	4.0±4.1	37.2±4.3	25.6±3.9	34.2±6.4	7.0±2.3	69.7±4.1
女性	74	34.5±5.8	11.9±4.0	40.5±5.8	21.6±4.8	35.8±5.7	13.5±4.0	56.6±5.8

*P*值		0.504	0.017	0.596	0.548	0.365	0.227	0.073

供受者性别							
相合	111	35.4±4.7	6.5±2.6	39.6±4.7	24.3±4.1	38.2±7.3	11.7±3.1	61.2±4.6
不相合	92	40.6±5.4	6.5±2.7	37.0±5.1	23.9±4.5	31.8±6.1	6.5±2.6	69.4±4.8

*P*值		0.416	0.932	0.694	0.958	0.446	0.133	0.180
								
供受者性别							
男供男	84	39.3±5.6	3.1±2.2	36.9±5.3	28.6±5.0	37.8±8.6	9.5±3.2	64.1±5.3
男供女	45	40.4±7.7	0.0±0.0	33.3±7.1	17.8±5.8	29.7±11.1	0.0±0.0	80.0±6.0
女供男	47	40.9±7.7	4.5±4.8	36.2±7.1	27.7±6.6	35.2±7.3	10.6±4.6	59.3±7.2
女供女	27	23.7±8.7	12.1±6.7	44.4±9.9	7.4±5.2	37.0±9.5	14.8±7.0	51.9±9.6

*P*值		0.547	0.095	0.710	0.186	0.563	0.147	0.068

ABO血型								
相合	113	38.4±4.8	9.2±3.2	36.3±4.6	26.5±4.2	29.9±5.0	8.0±2.6	67.0±4.5
次要不合	37	27.3±8.0	2.8±3.2	40.5±8.2	21.6±6.9	36.1±13.5	16.2±6.2	67.6±7.7
主要不合	39	51.3±8.5	0.0±0.0	41.0±8.0	20.5±6.6	42.8±8.4	5.1±3.6	58.9±7.9
主次均不合	14	22.9±11.9	0.0±0.0	28.6±12.7	7.1±7.1	35.7±13.4	7.1±7.2	57.1±13.2

*P*值		0.085	0.483	0.877	0.768	0.444	0.374	0.706

HLA相合位点								
5/10	153	37.6±4.1	8.4±4.1	36.6±3.9	22.9±3.4	39.0±8.0	7.8±2.2	65.8±3.9
6～7/10	34	49.8±9.1	0.0±0.0	44.1±8.7	29.4±8.0	44.7±8.9	5.9±4.1	55.9±8.5
8～9/10	16	12.5±8.6	7.4±2.7	37.5±12.6	18.8±10.0	6.3±6.3	25.0±11	75.0±10.8

*P*值		0.058	0.208	0.429	0.628	0.040	0.003	0.191

供者亲缘关系							
子女	108	27.2±4.4	7.4±2.7	34.3±4.6	13.9±3.4	43.4±8.4	7.4±2.5	59.8±4.8
父母	54	53.5±7.4	8.4±4.1	40.7±6.8	38.9±6.7	20.4±5.5	9.3±4.0	72.2±6.1
同胞或旁系	41	45.7±8.2	0.0±0.0	43.9±7.9	29.3±7.2	33.0±8.7	12.2±5.2	68.3±7.3

*P*值		0.007	0.208	0.332	0.001	0.110	0.603	0.332

**注** HLA：人类白细胞抗原；aGVHD：急性移植物抗宿主病；cGVHD：慢性移植物抗宿主病；CMV：巨细胞病毒；EBV：Epstein-Barr病毒；NRM：非复发死亡；OS：总生存。^a^Ⅱ～Ⅳ度aGVHD为100 d累积发生率，其他指标均为2年累积发生率

三、基于年龄及亲缘关系的亚组分析

为了确定性别和亲缘关系对移植预后影响，我们对不同年龄组分别进行了分析。

在供者年龄小于20岁的32例（16％）MDS患者中，单因素分析显示供者性别（男性对女性）对移植后2年OS率（71.3％对63.5％，*P*＝0.913）、2年无复发生存率（85.6％对92.9％，*P*＝0.716）及100 d内Ⅱ～Ⅳ度急性GVHD（22.2％对39.4％，*P*＝0.359）影响无统计学意义。在该亚组中，90.6％（29/32）供者为移植患者其子女。提示年龄<20岁的子女，无论是儿子或女儿作为供者，其移植预后并无显著差异。

20～50岁供者的移植患者占我们研究队列的74％（150/203）。与男性供者相比，女性供者移植后2年重度慢性GVHD的发生率更高（10.6％对1.3％，*P*＝0.022），其OS降低，但差异无统计学意义（56.7％对69.6％，*P*＝0.107），这与整体队列的结论一致。对该亚组供受者亲缘关系分析提示，子女单倍型供者，与同胞单倍型供者或父母单倍型供者相比，Ⅱ～Ⅳ度急性GVHD的累积发生率显著降低（27.9％对43.9％对61.2％，*P*＝0.008），EBV再激活率降低（16.5％对31.6％对33.3％，*P*＝0.061）。

年龄>50岁的供者共21例（10.3％），全部为父母单倍型供者。我们之前整体队列的研究已提示，年龄>50岁的供者移植后EBV激活风险增高，提示采用≤50岁的供者是更为理想的选择。然而，当患者缺乏≤50岁的供者时，50岁以上的父亲供者移植后2年OS率高于母亲供者，分别为66.6％和44.4％，差异无统计学意义（*P*＝0.170）。

## 讨论

近年来单倍型移植取得了快速发展，如何优化供者选择是临床面临的重要问题。我们报道的203例MDS单倍型移植的供者数据表明，供者年龄、性别及供受者亲缘关系是影响移植预后的重要因素。移植中避免采用50岁以上的供者。男性供者优于女性供者，子女单倍型供者优于同胞单倍型或父母单倍型供者。我们也分析了不同年龄亚组的供者特征对预后的影响，考虑病例数相对较少，仅对供者选择的优化提供思路，尚需要更大样本的研究证实。

既往有多项研究报道供者年龄对移植预后的影响：Wang等[14]研究表明，年龄<30岁的供者移植后有更好的OS且Ⅱ～Ⅳ度急性GVHD发生风险更低。Canaani等[15]分析了来自EBMT登记处的1 270例急性白血病患者接受单倍型移植的临床预后，结果表明当患者年龄>40岁时，供者年龄>40岁与≤40岁组相比，其OS、无白血病生存（LFS）及NRM明显更差。然而，当患者年龄<40岁，供者年龄>40岁时Ⅱ～Ⅳ度急性GVHD发生率显著增高，父母单倍型供者与同胞单倍型供者Ⅱ～Ⅳ度急性GVHD发生率分别为38％及28％（*P*＝0.017）。但如果供者年龄>55岁，患者发生重度慢性GVHD的风险反而降低（*HR*＝0.17，*P*＝0.045）。供受者亲缘关系的分析提示对于患者年龄>40岁，供者年龄>51岁提示患者累积复发率略有升高（*HR*＝1.82，*P*＝0.06），LFS亦有降低趋势（*HR*＝1.6，*P*＝0.057）。我们的结果同样证实年轻供者的优势，临床中尽量避免应用>50岁的供者。本研究中，我们选择50岁作为大龄供者临界值，主要基于MDS的老龄化趋势，此结果尚需要多中心研究进一步验证。此外，我们的研究将20岁以下供者作为单独的亚组分析，主要基于我国婚姻法规定的20岁为最低结婚年龄。20岁以下的供者均为未婚未育状态，避免妊娠对女性免疫状态的影响，从而明确年龄小于20岁的女性供者对移植的影响并不劣于年轻的男性供者。供者性别对预后的影响中，多项研究均发现男性供者的移植预后优于女性供者[14],[16]–[17]。我们的结果也证实女性供者移植后发生重度慢性GVHD风险增高，对OS产生不良影响。母亲供者移植后发生GVHD风险增高，这与Ma等[18]报道的结论类似，但具体的机制尚未完全清楚。

ABO血型不合对移植预后的影响已有报道，不同中心的结论有所不同。与Canaani等[19]报道结果类似，我们发现ABO血型不合与OS、NRM、复发率之间没有显著相关性。而亚组分析显示，供受者ABO主要血型不合，提示移植后OS较差。Logan等[20]提出，ABO血型次要不合与较差的OS和NRM相关，而在我们的研究中并未观察到这一点。Luger等[21]分析的MDS/AML患者移植中，ABO血型主要不合是OS和NRM的不良危险因素。而在淋巴瘤患者中并未观察到相似的情况[22]。另一项包括多种血液恶性肿瘤患者的移植中，发现ABO主要血型不合可引起移植后血小板重建延迟[23]。不同研究中关于ABO血型不合对移植后预后的不同报道，可能与病例纳入标准不同有关，例如疾病类型、供者类型、预处理方案等因素。

考虑到父母、子女、单倍型的同胞和旁系亲属都是潜在的供者，多项研究报道了供受者亲缘关系对临床预后的影响。关于供受者亲缘关系，我们的数据支持选择子女作为供者。因为与同胞供者或父母供者相比，子女供者移植后Ⅱ～Ⅳ度急性GVHD和EBV再激活的累积发生率均较低。由于供者的年龄、性别及亲缘关系均会影响移植效果，因此对这些因素的综合分析对于优化供者选择至关重要。尽管共识及多项研究均推荐年轻的男性供者为更优的选择[4],[9],[14]，但我们的研究表明，在年龄小于20岁的子女供者中，性别不影响移植预后，女儿或儿子供者都是合适的供者选择。

与既往研究存在的主要差异为我们的分析仅限于MDS患者这一单病种队列，而其他大部分研究均包含白血病在内的各种恶性血液病的混合队列。而且在移植预处理方案中，我们采用ATG体内去T的BUCY方案，而国外研究更多采用PTCY预处理方案。此外，本研究存在一定的局限性，基于研究数据均来自单中心回顾性分析，亚组分析涉及的病例数量相对不足，无法进一步进行多因素验证，因此对我们的结果应谨慎对待。也期望未来通过多中心研究，进一步扩大病例数量以解决这些问题。
